# A Bioinformatic Pipeline for Monitoring of the Mutational Stability of Viral Drug Targets with Deep-Sequencing Technology

**DOI:** 10.3390/v9120357

**Published:** 2017-11-23

**Authors:** Yuri Kravatsky, Vladimir Chechetkin, Daria Fedoseeva, Maria Gorbacheva, Galina Kravatskaya, Olga Kretova, Nickolai Tchurikov

**Affiliations:** Engelhardt Institute of Molecular Biology of Russian Academy of Sciences, Vavilov str., 32, Moscow 119334, Russia; jiri@eimb.ru (Y.K.); vladimir_chechet@mail.ru (V.C.); dfedoseeva86@yandex.ru (D.F.); maruseok@gmail.com (M.G.); gk@eimb.ru (G.K.); okretova@eimb.ru (O.K.)

**Keywords:** viruses, drug targets, mutations, deep-sequencing, data processing, bioinformatic pipeline

## Abstract

The efficient development of antiviral drugs, including efficient antiviral small interfering RNAs (siRNAs), requires continuous monitoring of the strict correspondence between a drug and the related highly variable viral DNA/RNA target(s). Deep sequencing is able to provide an assessment of both the general target conservation and the frequency of particular mutations in the different target sites. The aim of this study was to develop a reliable bioinformatic pipeline for the analysis of millions of short, deep sequencing reads corresponding to selected highly variable viral sequences that are drug target(s). The suggested bioinformatic pipeline combines the available programs and the ad hoc scripts based on an original algorithm of the search for the conserved targets in the deep sequencing data. We also present the statistical criteria for the threshold of reliable mutation detection and for the assessment of variations between corresponding data sets. These criteria are robust against the possible sequencing errors in the reads. As an example, the bioinformatic pipeline is applied to the study of the conservation of RNA interference (RNAi) targets in human immunodeficiency virus 1 (HIV-1) subtype A. The developed pipeline is freely available to download at the website http://virmut.eimb.ru/. Brief comments and comparisons between VirMut and other pipelines are also presented.

## 1. Introduction

Targeted gene therapy provides an efficient approach to the development of anti-bacterial and antiviral drugs. In such therapies, the drugs bind to specific targets within bacterial and viral proteins or genomes and thus suppress the activity of the pathogen [1,2,3]. However, mutations in DNA fragments corresponding to the drug targets destroy the binding specificity and produce so-called mutational escape from the particular drug. As mutations are often induced during replication, the rate of replication may affect the frequency of mutations. The mutational escape becomes especially significant for viruses that have relatively small genomes and high replication rates [4,5]. According to the World Health Organization, the annual global burden of communicable diseases (of which viral diseases play a major part) amounts to ~15 million cases [6].

The mutational stability of viral drug targets can be efficiently monitored by deep sequencing technologies [7,8,9,10,11,12]. Deep sequencing allows the rapid acquisition of thousands or millions of short nucleotide reads comprised of fragments corresponding to drug targets. These data allow detection of the conserved targets as well as the mutational repertoire that is typical for a particular patient or a cohort of patients. Following the detection of such targets, the conserved targets are used for testing the correspondence between the drug and its target and for the optimal choice of drug(s). The reliable assessment of drug-target stability against mutational escape needs very deep coverage of related DNA fragments corresponding to the drug targets (10^6^–10^8^ of reads), i.e., high-deep and ultra-deep sequencing. Most post-alignment deep sequencing tools are not designed for such deep coverage and cannot handle the data properly. Consequently, the processing of such massive data arrays requires the development of special bioinformatic methods and tools.

The processing of large data arrays related to deep sequencing is performed by bioinformatic pipelines [13,14,15]. A bioinformatic pipeline refers to an organized package of programs, such that the processed data at the output of one program are consecutively transferred to the input of another program for the subsequent processing. In this paper, we present a proposed bioinformatics pipeline based on the original scheme of the search for the conserved targets and including the original statistical criteria for mutational analysis with deep sequencing data. The application of the bioinformatic pipeline is illustrated by studying the conservation of RNA interference (RNAi) targets in human immunodeficiency virus 1 (HIV-1) subtype A [16,17,18]. Previously, we selected and analyzed six RNAi targets that perfectly meet the criteria for efficient small interfering RNA (siRNA) using real time PCR (RT-PCR), cloning, and sequencing. However, this approach provides only limited information about the variability in the RNAi targets. Therefore, we deep sequenced ≈100-bp regions that incorporated the selected RNAi targets. During the bioinformatic treatment of these data, we encountered a number of problems.

Comprehensive reviews on the application of bioinformatic pipelines for processing deep sequencing data related to drug-resistance in HIV can be found in [19,20]. For convenience, we have summarized the available pipelines in the Supplementary Materials and added brief comments on the utility of different packages.

After the selection and experimental trial of viral drug targets, their mutational conservation can be monitored by deep sequencing. The relevant data processing comprises searching for the most conserved targets in the complete set of reads and then the most conserved targets can be used as a reference for the assessment of mutation and indel frequencies. We have not found suitable available packages for the search and verification of conserved targets in the total set of reads and for subsequent representation of deep sequencing data in terms of mutation profiles together with the assessment of the statistical significance of these data. This motivated us to develop the pipeline presented below. Similar to the majority of available packages, our pipeline also operates in a semi-automatic mode.

## 2. Materials and Methods

### 2.1. Preparation of Libraries for the Deep Sequencing of RNA Interference Targets

**Figure 1:**
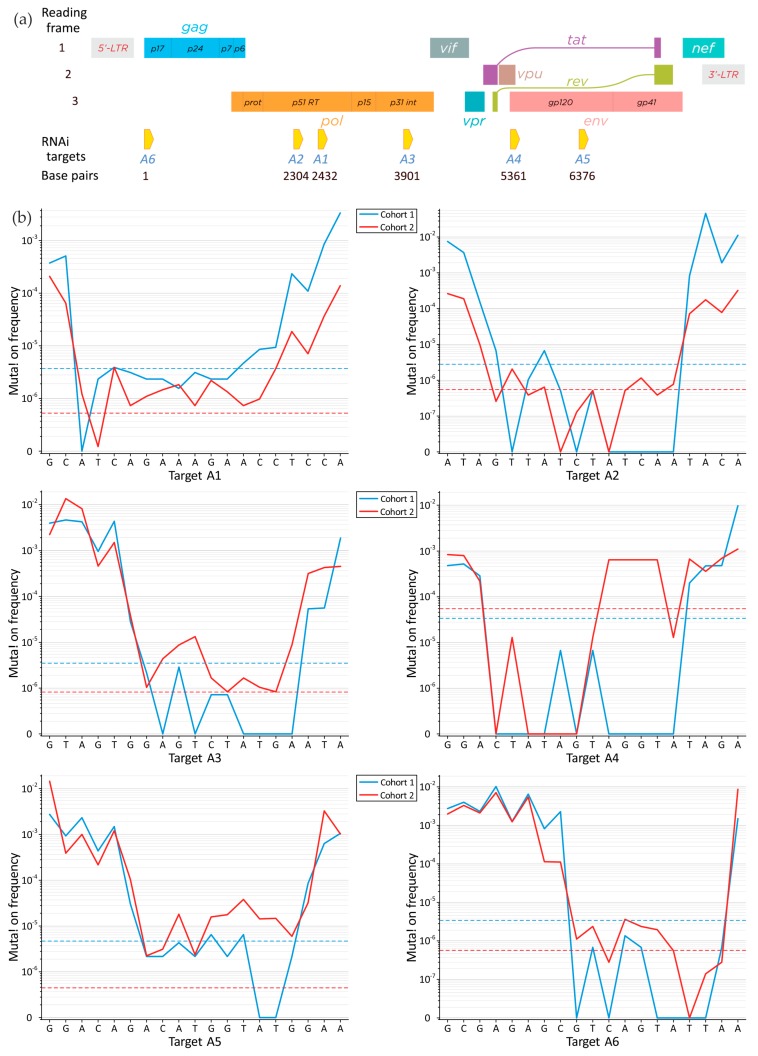
The positions of RNA interference (RNAi) targets on the genome of human immunodeficiency virus 1 (HIV-1) and their conservation within two independent cohorts of patients from Russia. (**a**) Schematic presentation of RNAi targets within the HIV-1 physical map; (**b**) The profiles of total mutation frequencies over the RNAi targets. The broken horizontal lines correspond to the thresholds of reliable mutation detection as determined by Equation (3).

We used two independent cohorts of patients from Russia. Cohort 1 included five isolates of HIV-1 subtype A from patients who were not receiving antiretroviral therapy. Cohort 2 included four isolates from patients that had received antiretroviral therapy for five years and still possessed high-titer viremia, and therefore, cohort 2 was considered to contain drug-resistant HIV-1 strains. Such cohorts can mimic the variability of RNAi targets for multi-strain HIV-1 patients and/or the development of multiple strains over time for a particular patient. For deep sequencing, we used the previously described six regions in the HIV-1 genome that incorporate efficient RNAi targets [16,17,18]. Regions of about 300 bp (A1–A6 regions in Figure 1a) were used for RT-PCR and preparation of the libraries for deep sequencing. The RNA preparations were provided by the State Research Center of Virology and Biotechnology Vector (Koltsovo, Novosibirsk Oblast, Russia) from their collection of isolates. Written informed consent was obtained from each patient. The RNA preparations were extracted individually from plasma samples and then the preparations from each cohort were pooled. About 15 ng of pooled RNA was used for RT-PCR. Moloney Murine Leukemia Virus (M-MLV) reverse transcriptase was used to synthesize complementary DNAs (cDNAs) using a DNA-free kit (Ambion, Foster City, CA, USA) according to the manufacturer’s instructions. Amplified DNA was used for the preparation of the library. Libraries were prepared according to the manufacturer’s instructions that accompany the DNA Sample Kit or the NEBNext Ultra DNA Library Prep Kit (Illumina, San Diego, CA, USA). Deep sequencing was performed using an Illumina Genome Analyzer IIx (Illumina) (cohort 1) or paired-end reads (2 × 150 bp) produced by an Illumina HiSeq 1500 (Illumina) (cohort 2). The data were deposited in the National Center for Biotechnology Information (NCBI, Bioproject Accession No. PRJNA344431).

### 2.2. Bioinformatic Pipeline and Data Processing

Here we describe the main stages of data processing within the bioinformatic pipeline. The raw sequenced reads were first evaluated for their quality using FastQC [21]. Then, the reads of too-short length (<20 nt) and of low quality (Q < 26) were filtered out from the initial set using cutadapt [22]. The threshold of Q was set to 26, which corresponds to the maximum of mapped reads [23]. The conserved targets that may subsequently be used as a natural reference for evaluation of mutation and microindel frequencies were determined using a two-step iterative procedure that is identical at both steps except that, during the first step, the procedure stops after the first 10,000 reads to check and refine the conserved target. If the current conserved sequence does not coincide with the initial reference sequence, then the reference sequence for alignment is replaced by the conserved sequence and the procedure restarts from the beginning. We have tested different deep sequencing aligners and chose Bowtie 2 in ‘local’ mode [24]. If the numbers of indels in an experiment would be significant, then replacing the Bowtie 2 by the subread aligner should be preferable, since the latter can control indel parameters [25].

At the first step, a target from HIV-1 isolate 97CDKP58e from the Republic of the Congo (GenBank Accession No. AF316544) was used as the reference for the first set of 10,000 reads using Bowtie 2 with option ‘very-sensitive-local’ [24]. All non-aligned reads were filtered out by SAMtools [26], whereupon all aligned reads were converted to Fasta format by SAMtools [26] and the seqtk toolkit. The aligners for monitoring of genetic variations with deep sequencing should provide the optimal speed and quality of alignment. To verify these claims, we used two-step processing. First, deep sequencing reads were aligned by Bowtie 2 to ensure a high processing speed. Then, the alignment obtained by Bowtie 2 was further processed by the slower pairwise Smith–Waterman algorithm, which ensures a high accuracy of alignment. In our ad hoc Perl script, we applied Smith–Waterman alignment from Fasta 3.5.4.12 package with options: -3 -E 0.01 -q -n. Fasta alignment parameters can be tuned according to individual experiments. To accelerate alignments, we used RAMdrive to store ‘database’ and ‘query’ files for Fasta. To identify the conserved target, the results of Smith–Waterman pairwise alignments were analyzed by our ad hoc Perl script. The sequence for the conserved target was refined and, at the second step, was used as the reference for the complete set of reads. In the subsequent re-run of the pipeline, the alignment relative to the refined reference was also performed by the Smith–Waterman algorithm and included the complete set of reads.

**Figure 2:**
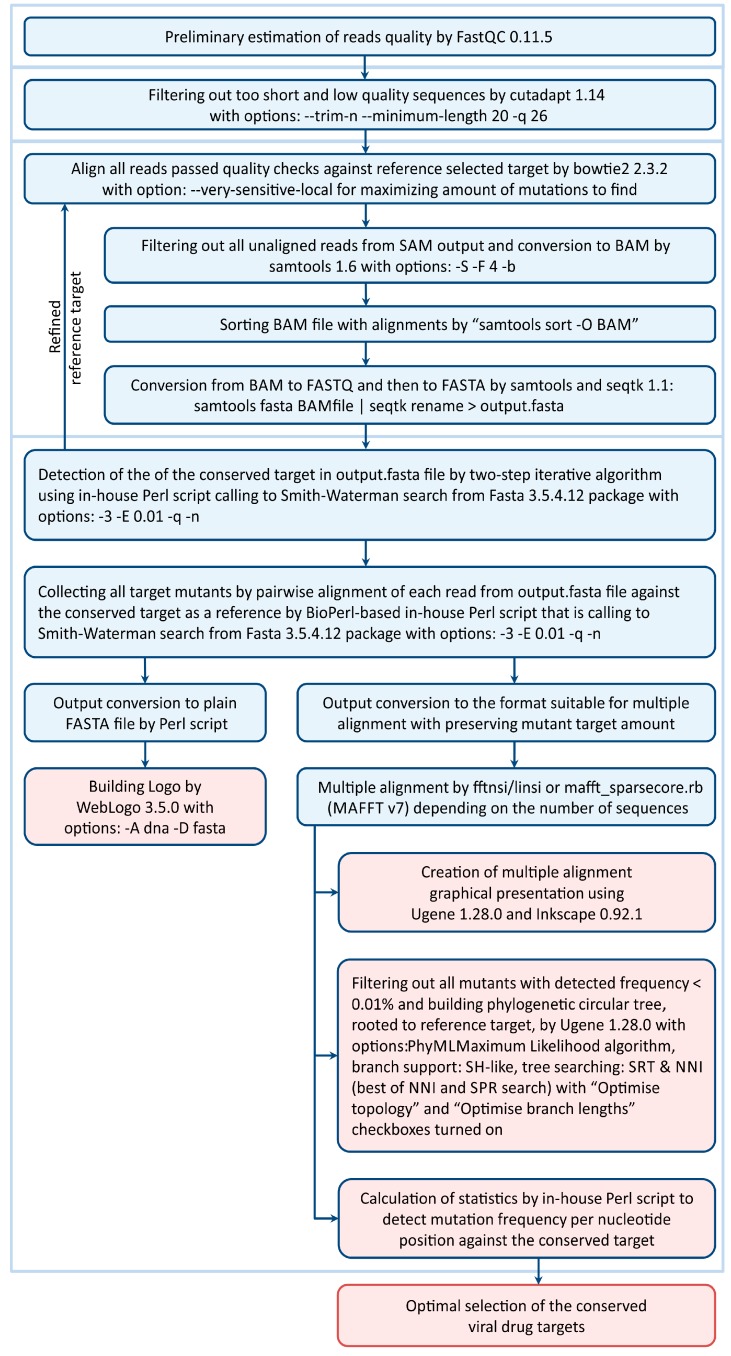
Scheme of the bioinformatics pipeline for deep-sequence monitoring of viral drug targets. The resulting output options are marked in red. The particular scripts correspond to the blocks of programs outlined in blue. The typical information related to the filtering and times for read processing is presented in Table S2. Links to all software used in the pipeline are given in Section 2.2.

At the second step, we obtained the full set of sequences that were pairwise aligned by the Smith–Waterman algorithm to the conserved target. Due to the huge quantity of reads, this set cannot be used directly for multiple alignment. Therefore, we used a compression of the total datasets as the clusters of the numbered multiple copies of unique sequences. This approach ensures a significant reduction in the set size for all potentially conserved reads and avoids any artificial statistical bias. As a result, we obtained a packed set with a total number of sequences that, in our case, did not exceed 20,000, which is suitable for multiple alignment by the available programs. By default, MAFFT [27] can process up to 40,000 unique sequences. In practice, the maximum number of processed sequences is limited only by the available RAM; for example, the number of sequences can be as high as 100,000 for 64 GB of RAM. The multiple alignment was performed using MAFFT software because it can achieve good alignment quality for large datasets typical of deep sequencing [27]. To analyze the results of multiple alignments and to calculate the corresponding mutation frequencies and related statistics, we developed a BioPerl-based script. This script has additional output options to visualize the results in terms of multiple alignments or phylogenetic trees using the Ugene toolkit [28]. We also developed a Perl script for the visualization of nucleotide substitution within target sites using WebLogo [29]. The blocks in the pipeline related to the particular scripts are individually outlined in Figure 2. To save space, we restricted the presentation of mutation profiles over targets in the Results section to the total frequency of mutations. Generally, the nucleotide substitution can be classified into all 12 types of replacements. The mutation frequencies averaged over the entire target region can be presented in terms of ranked histograms. The relevant results are illustrated in Supplementary Materials. We have created the website http://virmut.eimb.ru to support this work. It contains the bioinformatics pipeline diagram, as well as links to all the software used and pipeline source codes and a manual for installation and usage. The developed pipeline can be downloaded from the website where further comments and examples can be found.

## 3. Results

### 3.1. Search for the Conserved Targets and Statistical Criteria for Deep Sequencing Data Arrays

In the majority of bioinformatic pipelines (in fact, in all pipelines to the authors’ knowledge), the mutations are detected against fixed predetermined targets. The choice of such reference targets is partly dictated by the available drugs or merely by convention. For example, the sequences of HIV-1 isolate 97CDKP58e from the Republic of the Congo (GenBank Accession No. AF316544) could be considered as a generic reference for HIV-1 subtype A sequences. The conservation of a reference target and corresponding mutational repertoire that is determined against the complete deep sequencing set may depend on the choice of a particular target as a reference (see, for example, [17]). The natural choice of reference targets corresponds to the conserved targets in a set. Namely, these targets should be compared with related predetermined drug targets. Using the conserved target as a reference and aligning the complete deep sequencing set against such reference targets provides the frequency of nucleotide substitution and microindels in the different sites of the target and allows the assessment of the general conservation of the target.

The frequency of mutations, $f_{i,\;N\to N^{\prime }}^{\left(k\right)},$ is defined against the set of aligned sequences as

(1) $$f_{i,\;N\to N^{\prime }}^{\left(k\right)}=n_{i,N\to N^{\prime }}^{\left(k\right)}/n_{seq}^{\left(k\right)}$$

where $n_{i,N\to N^{\prime }}^{\left(k\right)}$ is the number of aligned sequences containing replacement $N\to N^{\prime }$ in a site *i* and $n_{seq}^{\left(k\right)}$ is the total number of aligned sequences for the *k*-th cohort. The total frequency of mutations in the site *i* is obtained by summation over $N^{\prime }\neq N$. The expected standard deviation for the mutation frequency in the *i*-th site may be assessed by binomial distribution [30,31]

(2) $$\sigma_{i,N\to N^{\prime }}^{\left(k\right)}={\left[f_{i,N\to N^{\prime }}^{\left(k\right)}(1-f_{i,N\to N^{\prime }}^{\left(k\right)})/n_{seq}^{\left(k\right)}\right]}^{1/2}$$

The similar expression is valid for the total frequency of mutations. The sensitivity of mutation detection with deep sequencing techniques can be assessed by the criterion $1.96\sigma (f_{thr})=f_{thr}$ (Pr = 0.05), which yields the threshold at a large *n_seq_*,

(3) $$f_{thr}\approx 3.84/n_{seq}$$

The detection limit (3) depends only on the total number of reads for a particular target and should be applied to the specific site in an individual target. The statistical significance between the corresponding replacements in the sites *i* and *j* for the same or two different cohorts can be assessed by the Gaussian *z*-criterion [30]

(4) $$z_{i|j,N\to N^{\prime }}^{\left(k|k^{\prime }\right)}=\frac{f_{i,N\to N^{\prime }}^{\left(k\right)}-f_{j,N\to N^{\prime }}^{\left(k^{\prime }\right)}}{{\left[f_{i|j,N\to N^{\prime }}^{\left(k|k^{\prime }\right)}(1-f_{i|j,N\to N^{\prime }}^{\left(k|k^{\prime }\right)})(1/n_{seq}^{\left(k\right)}+1/n_{seq}^{\left(k^{\prime }\right)})\right]}^{1/2}}$$

(5) $$f_{i|j,N\to N^{\prime }}^{\left(k|k^{\prime }\right)}=(n_{i,N\to N^{\prime }}^{\left(k\right)}+n_{j,N\to N^{\prime }}^{\left(k^{\prime }\right)})/(n_{seq}^{\left(k\right)}+n_{seq}^{\left(k^{\prime }\right)})$$

At $f_{N\to N^{\prime }}\approx 10^{-4}$ and *n_seq_* ≈ 10^6^, the difference about $\Delta f_{N\to N^{\prime }}\approx 10^{-5}$ can be resolved between the corresponding replacements $N\to N^{\prime }$ in two cohorts. In some cases, the small differences in mutation rates may have significant genetic consequences and may be used for the early diagnosis of disease.

All sequencing techniques introduce some experimental errors into the resulting reads. In Illumina deep-sequencing technology, the quality of reads is assessed in terms of the parameter *Q*, which is indirectly related to the error frequency. We will not discuss the possible methods for correction of the outputted Illumina data in this paper. Instead, we will restrict ourselves to the effects of given error rates on the proposed criteria and results. Let us consider a model in which the detected mutation frequency is composed of the actual mutation frequency and the frequency of read errors, *f*_observable_ = *f*_mutation_ + *f*_error_. Both contributions are inferred to be independent and to obey binomial statistics. The study of the cumulant generating function [30] for the composite stochastic process proves that, at the limit of small error frequencies, *f*_error_ << 1, the resulting statistics are approximately binomial or Gaussian. The inequality *f*_error_ << 1 is needed for the practical application of deep sequencing and is not restrictive. This means that the criteria (2)–(5) provide a suitable interpolation throughout the entire range of *f*_mutation_ when observable mutation frequencies are substituted into the criteria (2)–(5), i.e., these criteria remain robust against the contribution of the read errors.

Commonly, the noise in the experimental data (read errors in our case) is assessed via the weakest signals (detected mutation frequencies). The smallest detected mutation frequencies in the example below are about 10^−8^–10^−7^ and are several times (or even orders) less than the threshold related to the assessed statistical scattering in the finite sampling sets (see Equation (3)).

### 3.2. The Scheme of the Bioinformatic Pipeline

The general scheme of our bioinformatic pipeline is presented in Figure 2. The bioinformatics pipeline combines the available programs and the original scripts based, in part, on the original statistical methods. The relevant comments and details are given above in Section 2.2.

### 3.3. Mutations in RNA Interference Targets for HIV-1 Subtype A

**Figure 3:**
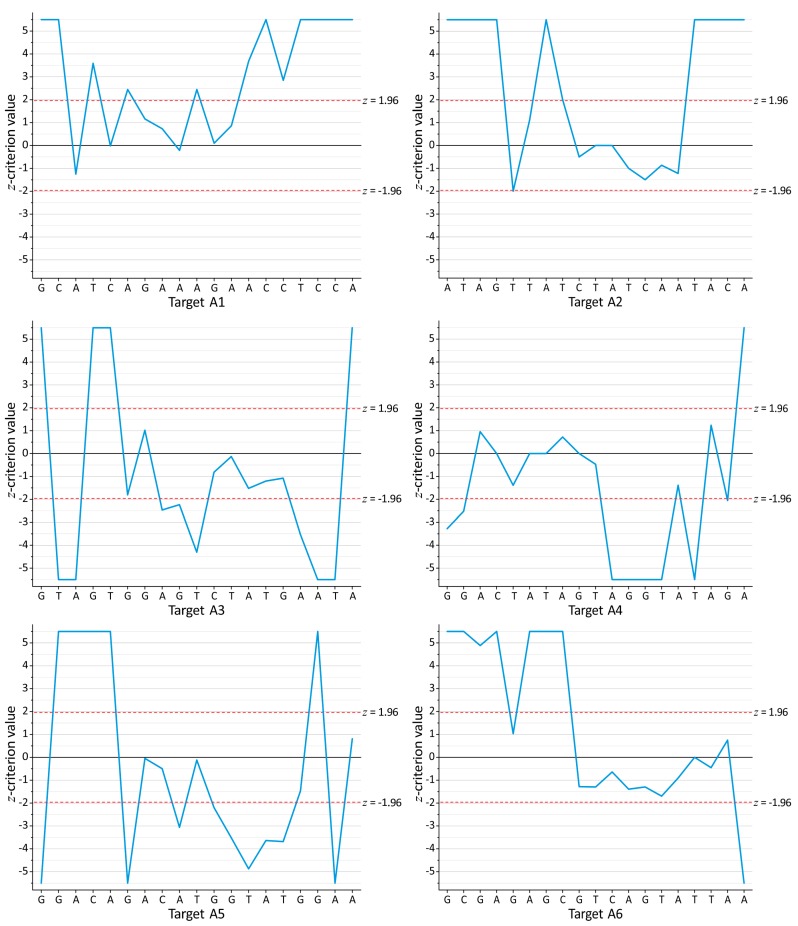
The *z*-criterion profiles (Equation (4)) characterizing the differences between mutation frequencies in the corresponding target sites for two independent cohorts of patients from Russia. For presentation purposes, the maximum absolute values of *z* were taken to be |*z*| = 5.5 (Pr = 3.9 × 10^−8^). The horizontal broken lines (*z* = ±1.96) correspond to the thresholds of statistical significance (Pr = 0.05).

A deep sequencing technique was applied for the study of the conservation of RNAi targets in HIV-1 subtype A. The detailed information about the selected RNAi targets A1–A4 and A6 was published previously [16,17]. The targets A1 and A2 are located inside the RT domain and A3 is inside the integrase domain, whereas A4, A5, and A6 reside inside the domains specifying vpu, gp120, and p17, respectively. Their positions on the HIV-1 genome are shown in Figure 1a. The total numbers of reads for the different RNAi targets are summarized in Table S1. The conserved targets were determined as described in the Methods section. Their 19-nucleotide core sequences are shown in Figure 1b together with the profiles of the total mutation frequencies over the target sites. The mutation profiles reveal the clear conservation of the target cores, thus indicating their functional significance. Rare microindels were also detected but their contribution to the general target conservation is about two orders of magnitude lower than that of mutations. The *z*-criterion profiles (Equation (4)) that characterize the difference between the mutation profiles for cohorts 1 and 2 in the corresponding sites (*i* = *j* in Equation (4)) of the same targets are shown in Figure 3.

### 3.4. Conservation of RNA Interference Targets for HIV-1 Subtype A

**Figure 4:**
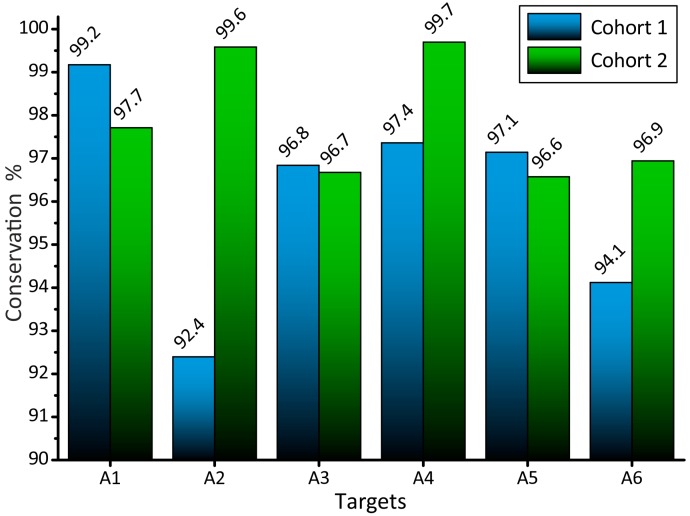
The conservation of RNAi targets (Equation (6)) for two independent cohorts of patients from Russia. The conservation of the targets is defined by Equation (6). The difference in the conservation of about 1% should be considered as statistically significant according to the Gaussian *z*-criterion.

The high specificity of drug-target recognition requires the strict conservation of targets. The conservation of a target is determined by the fraction of the conserved targets in the whole set of aligned sequences,

(6) $$I_{t}^{\left(k\right)}=n_{it}^{\left(k\right)}/n_{seq}^{\left(k\right)}$$

where $n_{it}^{\left(k\right)}$ is the number of the conserved targets for the *k*-th cohort. The conservation (Equation (6)) may also be treated as an empirical probability and used in the corresponding statistical estimates. The conservation of RNAi targets for HIV-1 subtype A in cohorts 1 and 2 is compared in Figure 4. The *z*-criterion for the difference in the conservation is defined in lines with Equations (4) and (5). Although the difference in the conservation between cohorts 1 and 2 seems to be rather small (about 1%), such a difference is highly statistically significant due to the large number of reads (about 10^6^) and thus may have genetic and medical relevance. For highly variable viral targets, the choice of the conserved targets is of primary importance.

## 4. Discussion

The conservation of viral drug targets assessed with deep sequencing provides useful quantitative criteria for the optimal choice of targets and relevant drugs. Such a technique may solve the problem of individual therapy because it is applicable to a particular patient. For the variable targets, one of the strategies for silencing virus activity consists of the multiplication of targets or using a combination of drugs. It was demonstrated that the multiplication of targets overcomes the mutational escape [16,17]. However, for practical purposes, a two-target combination is commonly sufficient.

The majority of available pipelines (reviewed in the Supplementary Materials) deals with fixed reference sequences and is not suitable for monitoring the mutational conservation of viral drug targets. They also do not include the options to generate the output data in terms of statistically significant mutation profiles and to refine the target under mutations automatically. The Segminator II package developed by Archer et al. [32] is the closest in terms of the aims and abilities of the pipeline presented in this paper. However, this package has not been updated since 2012 and now runs inadequately under modern Java and modern OSes, so its applicability cannot be tested; for example, GUI does not work properly after the alignment step. Additionally, Segminator II does not support modern paired-end sequencing data and, therefore, cannot be considered as an alternative to VirMut.

Deep sequencing with millions of reads may resolve small variations in mutation frequencies in the corresponding target sites for the different patients. Furthermore, ultra-deep sequencing with 10 million reads or more may resolve even smaller variations. The genetic and medical significance of the small variations in mutation frequencies is yet to be investigated even though the study looks promising. The monitoring of drug-target stability and the study of subtle effects cannot be performed without efficient bioinformatic toolkits. The processing of increasingly massive data arrays requires the development of increasingly sophisticated packages of programs or metaprograms, such as bioinformatics pipelines, workflow platforms, or cloud-based platforms.

The bioinformatics pipeline described here allowed us to identify the most conserved RNAi targets in the HIV-1 strains that, surprisingly, were found to be identical in up to 90% of viruses from both cohorts. The data could be used for the development of RNAi-based gene therapy of HIV/AIDS. We assume that, potentially, both chemically synthesized Dicer substrates (which are perfectly complementary to the detected RNAi targets in the viral transcripts) and genetic constructs (which express the biologically active siRNAs and are ex vivo-integrated into CD4+ T cells from a patient) could be used for such treatment. Moreover, combinatorial RNAi using two or more siRNAs targeting different targets can be used. This is important because a particular patient may possess HIV-1 strains with more than one conserved RNAi target and such multiplication of RNAi targets can be efficiently used for the suppression of HIV-1 activity [17].

Novel approaches using CRISPR/Cas9 gRNA-based (guide RNA) genome-editing have been developed for the permanent disruption of the HIV genome [33,34]. HIV-1 variability also hampers this approach because guide RNAs should be complementary to specific loci in the viral genome. Similar to the RNAi approach, it has been suggested that a combinatorial approach of two strong gRNAs targeting different regions of the HIV genome should be used [33]. Deep sequencing of the targets in HIV-1 strains is required for both RNAi and CRISPR/Cas9 approaches, which is why we believe that that the suggested bioinformatic pipeline will be useful in such studies.

## Supplementary Materials

The following are available online at www.mdpi.com/1999-4915/9/12/357/s1. Table S1: The total number of reads for RNAi targets in cohorts 1 and 2; Table S2: The processing time and number of reads passed each step of VirMut for the target A3 neighborhood; Figure S1: Circular PhyML tree for the target A1 in the cohort 1 rooted to the reference sequence corresponding to the conserved target in this cohort; Figure S2: Logo representation of target A1, cohort 1; Computer code: bioinformatic pipeline sources and installation guide; Review S3: Brief review of the bioinformatic pipelines for monitoring human immunodeficiency virus resistance with deep sequencing.
