# Supplementary material for: A Bioinformatic Pipeline for Monitoring of the Mutational Stability of Viral Drug Targets with Deep-Sequencing Technology
Source: Viruses. 2017 Nov 23;9(12):357. doi: 10.3390/v9120357 (PMC5744132; doi:10.3390/v9120357)
Supplement: Supplementary file 1 [file viruses-09-00357-s001.pdf]

Table S1. The total number of reads for RNAi targets in cohorts 1 and 2 at the input and output of data processing by the VirMut pipeline.

Number of reads				
RNAi target	Cohort 1 input	Cohort 1 output	Cohort 2 input	Cohort 2 output
A1	2,628,172	1,003,433	12,294,572	8,737,251
A2	3,989,469	1,963,858	11,781,904	8,622,507
A3	2,621,018	1,118,344	9,255,969	6,923,679
A4	1,416,229	119,605	13,178,914	1,382,778
A5	2,388,885	927,616	12,306,683	8,854,104
A6	3,669,638	995,520	12,314,155	9,666,188

The data correspond to the filtered reads that were subsequently used for the analysis of mutations and for the assessment of the general conservation of RNAi targets.

Table S2. The processing time and number of reads passed each step of VirMut for the Target A3 neighborhood.

VirMut steps	Time, h.m.s.	Time, %	Sequences	Sequences, %
0qualcheck.sh	0 min 30 sec	0.521	9,265,038	100.0
1qualtrim.sh	0 min 30 sec	0.521	9,255,969	99.9
2align.sh	0 min 52 sec	0.903	8,687,370	93.8
3stat.sh	1 h 34 min	98.055	6,306,312	68.1
Total	1 h 36 min	100.0		

Benchmarking was performed using an eight-core 64Gb RAM computer.

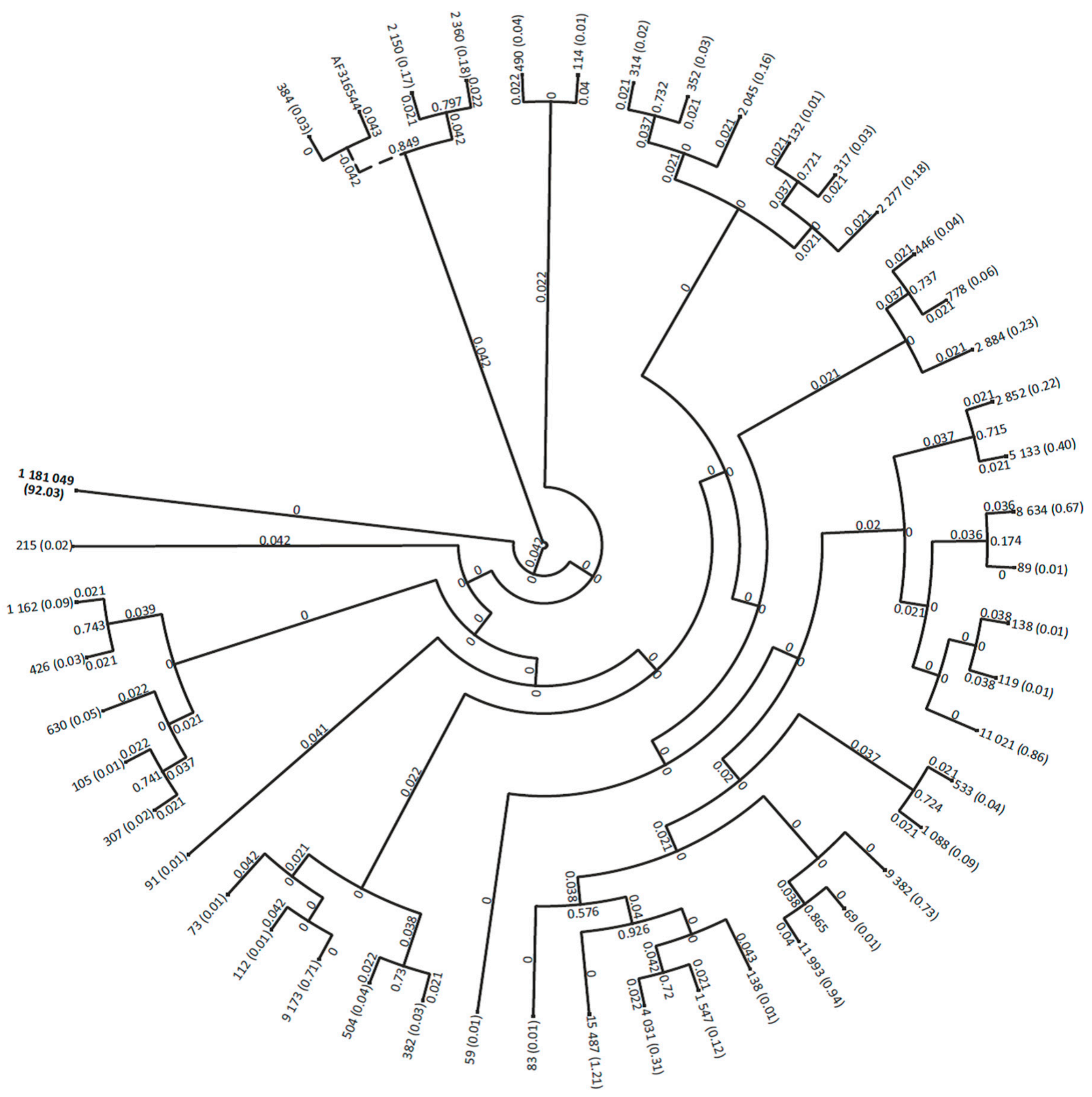

Figure S1. Circular PhyML tree for the target A1 in the cohort 1 rooted to the reference sequence corresponding to the conserved target in this cohort.

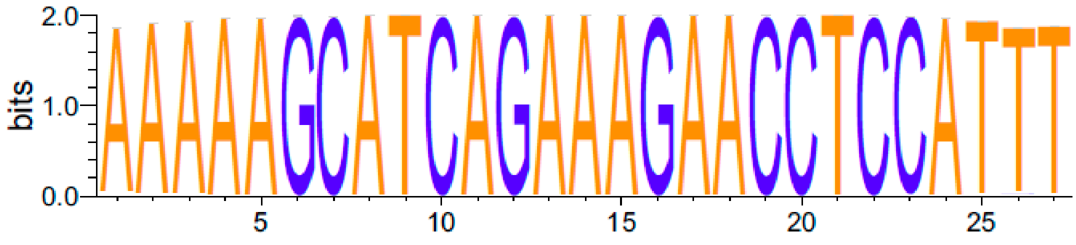

Figure S2. Logo representation of target A1, cohort 1.

Review S3. Bioinformatic Pipelines for Monitoring of Human Immunodeficiency Virus Resistance with deep sequencing.

HyDRA: https://hydra.canada.ca/
Program type: Web-interfaced pipeline (does not require user computer resources).
Program is aimed at identifying HIV drug resistance from a deep sequencing dataset. It can test user's sample versus Stanford Sierra HIV DB or custom mutation database created by user.

The potential users: Clinicians.
Commercial use: No.
Bioinformatic skills required: From none (using default values) to minimal (to tune thresholds and error rates).
Virus species: HIV-1 only (subtype cannot be chosen).
Customizability: Mutation database can be created and uploaded for analysis. Thresholds and error rates can be customized. Reference genome cannot be changed. Pipeline components cannot be changed/replaced.
Cannot be run locally as a single program at researcher's computer. Source codes not available.
Cannot be used for testing drug targets like RNAi targets for mutation frequencies.
Cannot generate mutation profiles in user's supplied dataset.

PASeq: https://www.paseq.org
Program type: Web-interfaced pipeline (does not require user computer resources).
Program is aimed at HIV resistance genotyping using deep sequencing data. It can test user's data set versus Stanford HIV DB.
The potential users: Clinicians.
Commercial use: No.
Bioinformatic skills required: None.
Virus species: HIV-1 only (subtype can be chosen).
Customizability: Does not support user's mutation database. Reference genome cannot be changed. Pipeline components cannot be changed or parameters tuned.
Cannot be run locally as a single program at researcher's computer. Source codes not available.
Cannot be used for testing drug targets like RNAi targets for mutation frequencies.
Cannot generate mutation profiles in user's supplied dataset.

V-Phaser 2 https://www.broadinstitute.org/viral-genomics/v-phaser-2
Program type: Linux command-line pipeline component.
Program is aimed at the study of variations in genetically heterogeneous populations from deep sequencing data. It combines information concerning covariation between observed variants to increase sensitivity and an expectation maximization algorithm that iteratively recalibrates base quality scores to increase specificity. V-Phaser can reliably detect rare variants in diverse populations that occur at frequencies of <1%.
The potential users: Bioinformaticians-programmers who create pipelines.
Commercial use: No.
Bioinformatic skills required: Professional in bioinformatics and Linux to create pipeline, professional programmer's skills to customize V-Phaser 2.
Virus species: Any (supplied by user).
Customizability: Any (C/C++ programming skills are required).
Should be run locally at researcher's computer (or at his cluster/cloud). Source codes available.
Cannot generate mutation profiles in user's supplied dataset.
Can be used in VirMut pipeline to determine consensus.

MiCall: https://github.com/cfe-lab/MiCall
Program type: Linux command-line pipeline.
Program is aimed at mapping all reads from a sample against a set of reference sequences and subsequent concatenating all reads into consensus sequences and coverage maps. It can test user's sample versus Stanford Sierra HIV DB or custom mutation database created by user. Pipeline has built-in monitoring system that regularly checks the file system for unprocessed runs, transfers input files to the cluster, and executes the pipeline (production mode).
The potential users: Bioinformaticians, researchers (if pipeline is preinstalled and configured by bioinformatician).
Commercial use: No.
Bioinformatic skills required: high.
Virus species: Any (supplied by user).
Customizability: Any (Python and Linux shell programming skills are required).
Pipeline components can be changed/replaced, any parameters can be tuned.
Mutation database can be created and used for analysis.
The pipeline should be run locally at researcher's computer (or at his cluster/cloud). Cannot be run locally as a single program at researcher's computer (MiCall is the set of scripts that invokes external aligner and BAM-files processing tools). Source codes are available.

Cannot be used for testing drug targets like RNAi targets for mutation frequencies.
Cannot generate mutation profiles in user's supplied dataset.
Some components of MiCall can be used in VirMut pipeline to map a sample set to reference sequence and determine consensus.

MinVar https://ozagordi.github.io/MinVar/
Program type: Linux command-line pipeline.
Program is aimed at search for drug resistance mutations in HIV-1 populations using deep sequencing data. It can test user's sample versus Stanford Sierra HIV DB. Mutations above 1.5% frequency are reported, but it is argued that mutations below 5% should be treated with caution.
The potential users: Clinicians.
Commercial use: No.
Bioinformatic skills required: None (to use), Linux administrator (to install).
Virus species: HIV-1.
Customizability: Any (Java and Linux shell programming skills are required).
Pipeline components can be changed/replaced, any parameters can be tuned.
Mutation database can be created and used for analysis after pipeline customization.
The pipeline should be run locally at researcher's computer (or at his cluster/cloud). Cannot be run locally as a single program at researcher's computer (MinVar is the set of scripts that invokes external aligner and BAM-files processing and filtering tools). Source codes available.
Cannot be used for testing drug targets like RNAi targets for mutation frequencies.
Cannot generate mutation profiles in user's supplied dataset.
Can be used in VirMut pipeline to determine consensus.

Segminator II http://www.bioinf.manchester.ac.uk/segminator/
Program type: Integrated Java all-in-one application with Graphical user interface.
Program is aimed at processing and downstream analysis of deep sequencing short-read viral deep sequencing data.
It can perform the seamless mapping and aligning of read data to a reference sequence; the association of multiple datasets to a single reference sequence; the per site coverage, entropy and base frequencies with the ability of particular site(s) query; the generation of consensus sequence and the assessment of related codon frequencies; phylogenetic inference; read visualization and a Bayesian framework for platform error correction.
The potential users: Researchers, bioinformaticians.
Commercial use: No.
Bioinformatic skills required: Low.
Virus species: Any (supplied by user).
Customizability: Thresholds and error rates can be customized.
Should be run locally at researcher's computer as a single program. Source codes are not available.
Does not support pair-end sequencing data sets.
Does not support pre-alignment processing and quality trimming.
Does not support reference sequence refining.
The program runs under modern Java and modern OS (no updates since 2012) inadequately, so applicability cannot be tested (GUI does not work properly after alignment step).

VirMut: http://virmut.eimb.ru
Program type: Linux command-line pipeline.
Program is aimed at the assessment of mutation and indel frequencies in viral drug targets with deep sequencing.
The potential users: Researchers, bioinformaticians.
Commercial use: No.
Bioinformatic skills required: from low to medium.
Virus species: Any (supplied by user).
Customizability: Any (Perl and Linux shell programming skills are required).
Pipeline components can be changed/replaced, any parameters can be tuned.
The pipeline should be run locally at researcher's computer (or at his cluster/cloud). Cannot be run locally as a single program at researcher's computer (VirMut is the set of scripts that invokes external aligner and BAM-files processing tools). Source codes are available.

Can be used for testing conservation of viral drug targets like RNAi targets and for assessment of mutation frequencies.
VirMut supports reference sequence refining.
Can generate mutation profiles in user's supplied dataset.
Supports multiple alignment creation.
